# Optimization of Causative Factors for Landslide Susceptibility Evaluation Using Remote Sensing and GIS Data in Parts of Niigata, Japan

**DOI:** 10.1371/journal.pone.0133262

**Published:** 2015-07-27

**Authors:** Jie Dou, Dieu Tien Bui, Ali P. Yunus, Kun Jia, Xuan Song, Inge Revhaug, Huan Xia, Zhongfan Zhu

**Affiliations:** 1 Department of Natural Environmental Studies, The University of Tokyo, Kashiwa, Japan; 2 Geographic Information System Group, Department of Business Administration and Computer Science, Telemark University College, Telemark, Norway; 3 State Key Laboratory of Remote Sensing Science, School of Geography, Beijing Normal University, Beijing, China; 4 Center for Spatial Information Science, University of Tokyo, Kashiwa, Japan; 5 Department of Mathematical Sciences and Technology, Norwegian University of Life Sciences, Ås Norway; 6 Guizhou University of Finance and Economics, Guiyang, China; 7 College of Water Sciences, Beijing Normal University, Beijing, China; University of New England, AUSTRALIA

## Abstract

This paper assesses the potentiality of certainty factor models (CF) for the best suitable causative factors extraction for landslide susceptibility mapping in the Sado Island, Niigata Prefecture, Japan. To test the applicability of CF, a landslide inventory map provided by National Research Institute for Earth Science and Disaster Prevention (NIED) was split into two subsets: (i) 70% of the landslides in the inventory to be used for building the CF based model; (ii) 30% of the landslides to be used for the validation purpose. A spatial database with fifteen landslide causative factors was then constructed by processing ALOS satellite images, aerial photos, topographical and geological maps. CF model was then applied to select the best subset from the fifteen factors. Using all fifteen factors and the best subset factors, landslide susceptibility maps were produced using statistical index (SI) and logistic regression (LR) models. The susceptibility maps were validated and compared using landslide locations in the validation data. The prediction performance of two susceptibility maps was estimated using the Receiver Operating Characteristics (ROC). The result shows that the area under the ROC curve (AUC) for the LR model (AUC = 0.817) is slightly higher than those obtained from the SI model (AUC = 0.801). Further, it is noted that the SI and LR models using the best subset outperform the models using the fifteen original factors. Therefore, we conclude that the optimized factor model using CF is more accurate in predicting landslide susceptibility and obtaining a more homogeneous classification map. Our findings acknowledge that in the mountainous regions suffering from data scarcity, it is possible to select key factors related to landslide occurrence based on the CF models in a GIS platform. Hence, the development of a scenario for future planning of risk mitigation is achieved in an efficient manner.

## Introduction

The Osado Mountain that runs through the Osado region, is a part of Sado Island, Niigata Prefecture, and stretches approximately 20 km off the north-western coast of the Honshu Island in the Sea of Japan. Over a long period of time, the stormy waves of the Japan Sea have produced cliffs and bizarrely cut rocks all around the Osado coasts. Geologically, the region is known for its history of gold and silver mining that started way back in the 8^th^ century. Natural hazards such as earthquakes or volcanic eruptions are rare compared to other islands of Japan, however, landslides have been common in the Osado mountain due to rugged topography and high elevation up to 1172 m [1]. Due to the low population density on Sado Island, effects of landslides on lives and properties are limited. The population of Sado Island has fallen from 125,597 in 1950, to 63,231 in 2011, representing more than a 45% decrease in the last 60 years [2]. However, landslides can cause problems to natural ecosystems and road networks in the Osado region. Therefore, it is necessary to assess the areas susceptible to landslides in order to mitigate damages associated with them.

Landslide susceptibility maps (LSM) play a vital role in assisting and managing hazards for land use planning and risk mitigation [3–6]. LSM provide information on the likelihood of landslides occurring in an area given the local terrain conditions [7]. Using GIS, various methods for landslide susceptibility mapping have been proposed in the past. These methods can be grouped into qualitative and quantitative, based on the properties they involve[8,9]. Qualitative methods denote susceptibility levels in descriptive terms using expert knowledge [10]. Such techniques are relatively subjective and were extensively used during 1970s and 1980s [11,12]. A main limitation of qualitative method is that the accuracy depends on the knowledge of the experts who conducts the research. Quantitative methods, on other hand investigates the relationship between landslide and causative factor to predict the occurrence probabilities [13,14]. Compared to the former one, a more realistic susceptibility map can be obtained from statistical and numerical methods [3] since they reduce the subjectivity and biases in the process of weighting landslide causative factors.

A wide range of quantitative methods have been successfully used for landslide susceptibility mapping by researchers around the globe [3,15,16]. The widely used methods are bivariate, multivariate [3,17], and logistic regression (LR) [10], neuro-fuzzy [18,19], support vector machines [19–21], and probabilistic models using Monte Carlo simulation with GIS [22,23]. The bivariate and multivariate statistical methods estimate landslide probabilities based on correlation analysis between causative factors and historical landslide events, whereas the deterministic methods assess slope failures using the factor of safety (FoS) [24,25]. In literature, statistical index (SI) and LR are considered to be the most commonly used methods for the assessment of probability of occurrence of landslides at medium and regional scales [26,27]. In contrast, FoS is used widely for the landslide assessment at large scales [8,16,28]. The advantage of LR over other multivariate analysis methods is that it is independent on data distribution and can handle a variety of data sets such as continuous, categorical, and binary data [3,29]. However, if a set of irrelevant independent variables are included, the LR model may have little to no predictive value. Owing to such constraints, prediction of landslide susceptibility requires a distributed approach that identifies all the relevant independent aspects of models used. In addition to that, successful landslide susceptibility mapping require optimal causative factors as input to the LSM models. In landslide studies, causative factors are usually selected based on the analysis of the landslide types and the characteristics of the study area [1]. Commonly used causative factors are elevation, slope angle, slope aspect, plan curvature, and distance to drainage networks [15]. However, most scholars randomly and subjectively selected the causative factors such as geological, geomorphological, hydrological and anthropogenic factors to produce the landslide susceptibility maps.Hence, selection of landslide causal factors and their classes are key points in LSM studies [27,30]. Lee and Talib (2005) [31] noted that the selection of positive factors can improve the prediction accuracy of the LSM. This indicates that the optimized factors are significant to LSM. Thus, before building the susceptibility models, predictive abilities of the initial selected factors should be quantified and factors with very low or null predictivity should be removed. This helps to reduce noise and uncertainties and thus the prediction ability of the resulting models will improve [32]. For instance, Pradhan and Lee (2010) [28] removed the causative factors with small weights and trimmed down the original factors to smaller numbers viz four, seven and eleven. Their research concluded that seven factors gave the best predicting accuracy. However, it is difficult to decide the threshold of weight to select the causative factors. Lee and Talib (2005) [31] used factor analysis method to remove the correlated variables, however it is a time-consuming process. Jebur et al. (2014) [33] followed an optimization technique for detecting best landslide causative factors, but their methods require preparation of multiple factor sets which is again time consuming. Although various other techniques have been proposed such as linear correlation [34], Goodman–Kruskal’s gamma [30], and GIS matrix combination [35], no standard guideline is available to date. Here, we address this issue by proposing the Certainty Factor method that has rarely been used for feature selection in landslide studies [36]. CF is an approach using rule-based expert systems to resolve certain problem classes. In the past, the search for the probabilistic interpretation of CF model has received considerable attention from the scholars [16,36–38]. In this study, CF is applied for selecting the positive causative factors related to landslide occurrence. Compared with the other methods, CF can be relatively easy to perform when having to integrate different layers using the combination rule [16,36,38].

**Fig 1:**
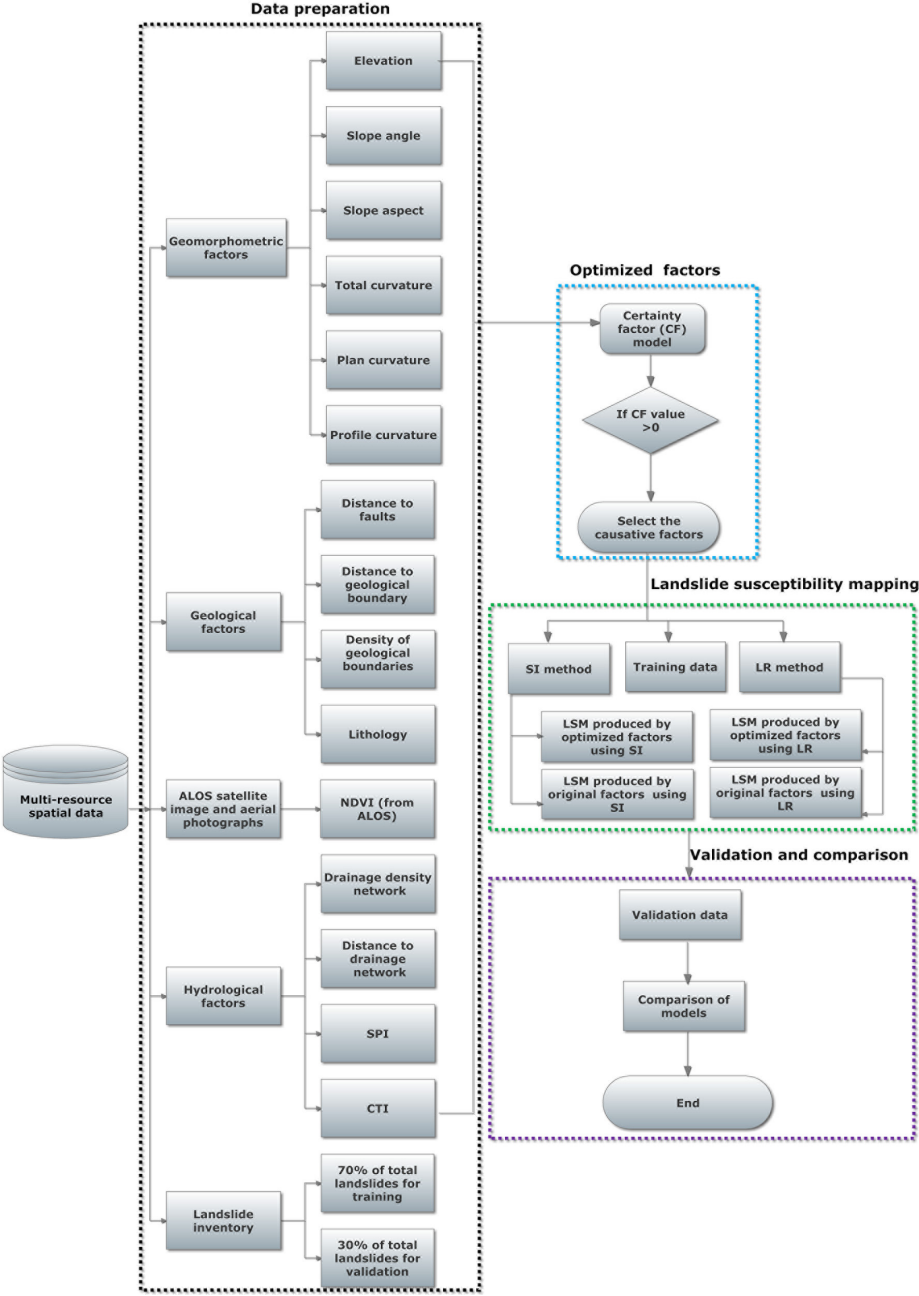
Flowchart shows overall methodology adopted for this study.

In Sado Island, the landslides are triggered mostly by rainfall and partly by snow melting [1,15]. The dominant lithologic units in Sado Island are volcanic dacite and andesitic lava, with rhyolitic intrusive at few locations (Geological Survey of Japan). Ayalew et al. (2005) [1] reported a high frequency of landslide occurrences in the ridges of the Kosado Mountain, a mountain range that runs parallel to the Osado Mountain. Geologically and topographically, both mountain ranges are similar. Hazard maps are available for Kosado Mountain from earlier researches that were prepared using semi-qualitative methods and pre-defined set of causative factors [1]. The outline of the present research paper is shown in Fig 1: (i) Data preparation and extraction of the landslide causative factors; (ii) Selection of the best subset of the causative factors using the CF method; and (iii) Landslide susceptibility mapping using the SI and LR method, (iv) Model validation and comparison.

## Study Area and Spatial Data

### 2.1 Topographical and geological settings in the study area

**Fig 2:**
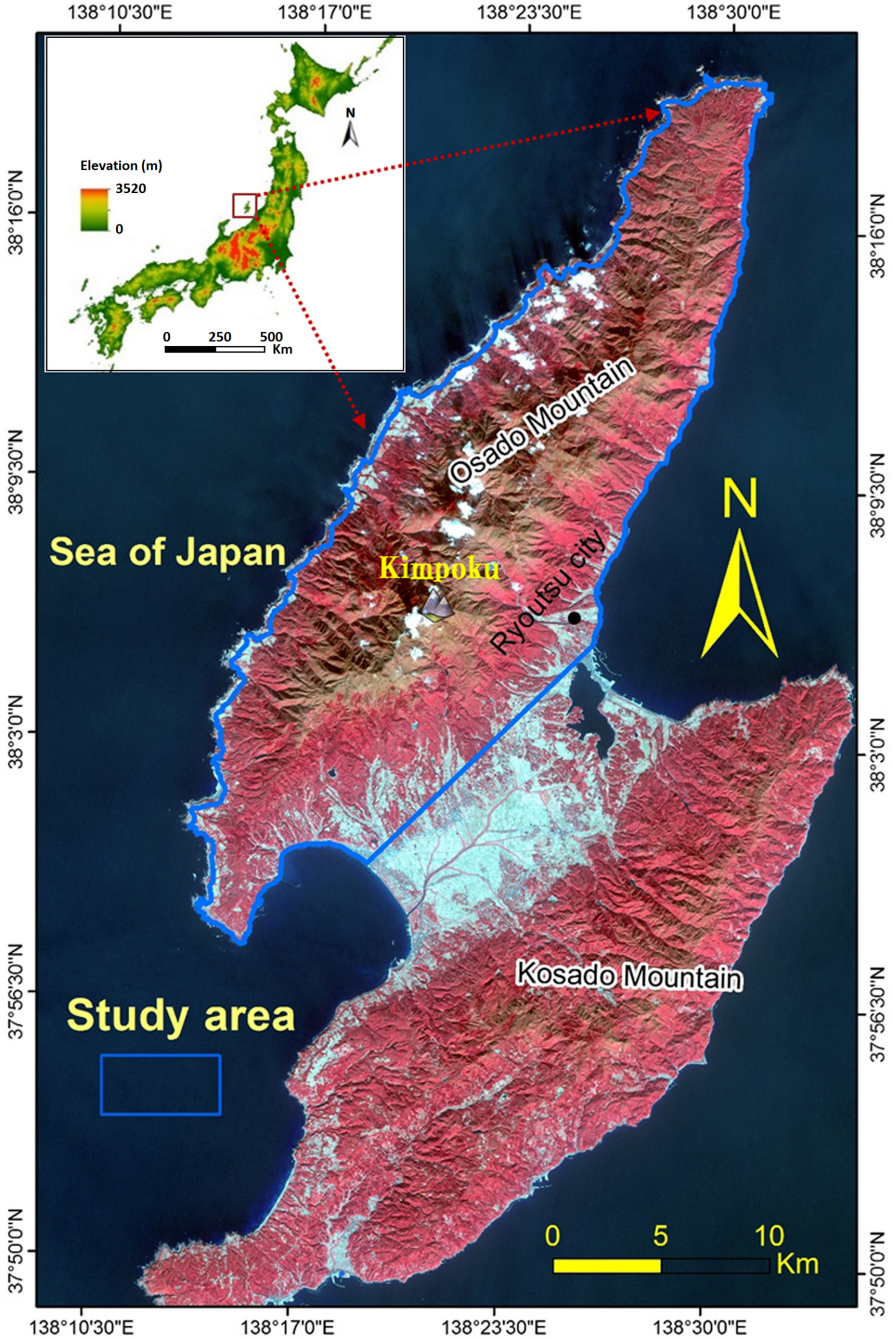
False color composite 3D view of the study area prepared from ALOS image.

The study area (Fig 2) is located in Sado Island, Niigata Prefecture, Japan, between longitudes 138°14'-138°32'E, and latitudes 37°57'-38°20'N. It covers an area of nearly 400 km^2^, mostly covered by vegetation. Vegetation in red color shown in the Fig 2 reflects high reflectance in the Near-IR Advanced Land Observing Satellite (ALOS) images. The elevation varies from 0 to 1172 m a.s.l with a mean of 333 m a.s.l. The highest peak of the island is the Mt. Kimpoku in the Osado Mountains. The geology is composed of Neogene marine volcanic sediments of dacitic and andesitic composition, associated with pyroclastics and rhyolitic intrusives in green tuffs. Some coastal slopes involve lately formed semi-consolidated and unconsolidated sand deposits and gravel. This area is frequently prone to landslides and subjected to tectonic movements that are evidenced by thrust up benches and active faults. The landslides in the study area are mostly deep-seated landslides and occasionally shallow landslides, which are triggered by rainfalls and snow-melt floods.

### 2.2 Landslide inventory

**Fig 3:**
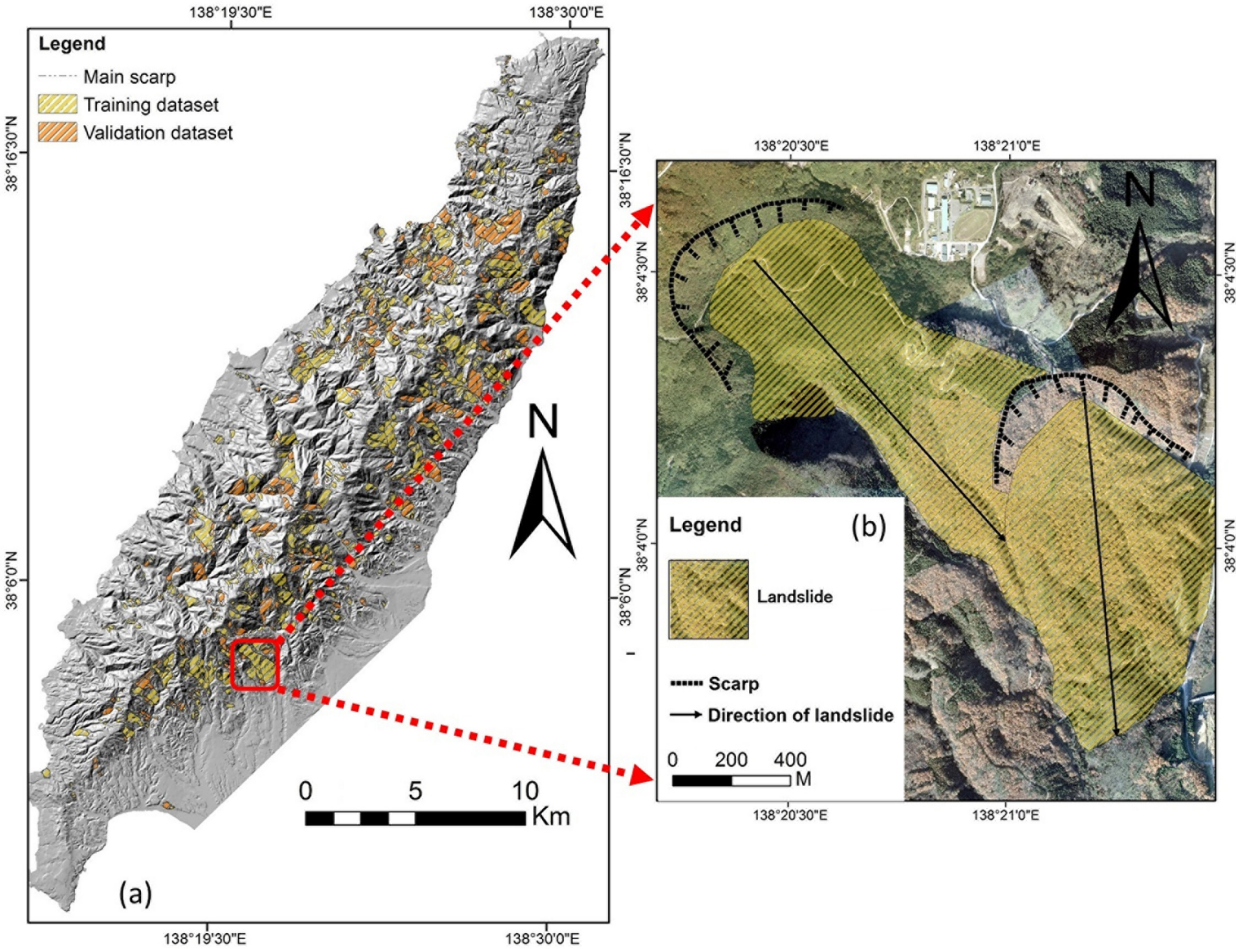
(a) Landslide inventory map for the study area randomly divided into two groups overlaid the shaded relief (10 m DEM): training dataset and validation samples: (b) enlarged view of boxed area in (a) overlaid on 2005 aerial photographs provided by the Midori Niigata and Sado city acquired in 2005.

**Fig 4:**
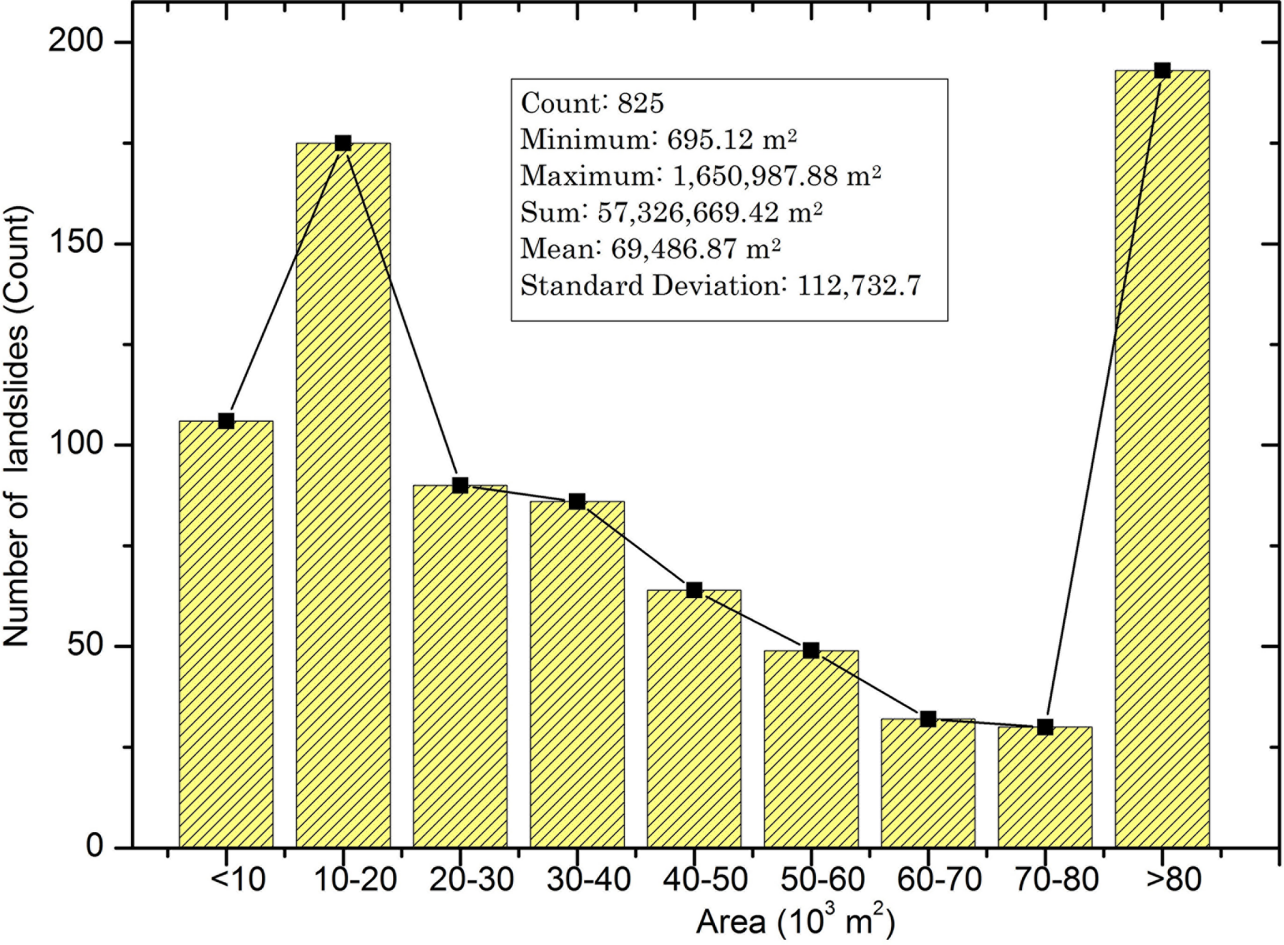
Histogram showing the distribution of landslide sizes.

According to Guzzetti et al. (1999) [39], landslides which occurred in the past and present are keys to predicting landslides happening in future. Hence, the first step in landslide susceptibility investigation is to compile the known landslide inventories. The details of the data used in this study are itemized in Table 1. A total of 825 known landslides (Fig 3) was first obtained for the model development; these landslides were interpreted by the landslide experts at the National Research Institute for Earth Science and Disaster Prevention (NIED), Japan. NIED has been producing landslide inventories since the year 2000 from the repeated acquisition of multiple aerial photographs. The landslides are depicted as boundary polygons in GIS shape file format and are available at NIED archives for the end users (http://lsweb1.ess.bosai.go.jp/gis-data/index.html). The archived landslide inventory database were also used in the previous researches to produce successful landslide hazard map in other study regions [40]. It is observed from the landslide inventory map that most landslide areas are greater than 0.01 km^2^. The minimum area observed is 0.0006 km^2^, whereas the largest landslide covers an area of about 1.65 km^2^ (Fig 4). The total area of landslides are about 57 km^2^, and accounts for approximately 15% of the study area.

**Table 1:** List of data sources used in the study.

Spatial database	Data	GIS/RS data type	Scale / Resolution	Data source
**Landslide inventory map**	Landslide	Polygon coverage	1:50,000	NIED
**Geological map**	Lithology	Polygon coverage	1:200,000	GSJ
	Faults	Line coverage		
	Geological boundary	Line coverage		
**Topographic maps**	Morphometric factors	ARC/INFO Grid	10×10 m	GSI
**ALOS**	NDVI	Raster	10×10 m	JAXA
**Aerial photographs**	Landslide direction	Raster	0.25×0.25 m	Midori Niigata and Sado City

Different sampling strategies are available to construct the reliable landslide susceptibility maps. Several previous researches preferred to use ‘points’ to represent the spatial location of landslides [13,19]. Dai and Lee (2003) [41] delineated only the source areas during the landslide susceptibility assessments and excluded both the transport and the deposition zones of existing landslides. Few other studies preferred to use the landslide area with depletion and accumulation zones like “seed cells” to represent pre-failure conditions [40,42,43]. Seed cells are the zones that are regarded to represent the undisturbed morphological condition [43]. Comparisons of these sampling strategies are however beyond the scope of this study. Here, we adopted one of the most popular method, the polygon of landslide to represent the spatial location [3,9]. For building the CF based LSM models, the landslide inventory was randomly partitioned into two groups: a training dataset (70%, 578 landslides) and a validation dataset (30%, 247 landslides).

### 2.3 Landslide causative factors

**Fig 5:**
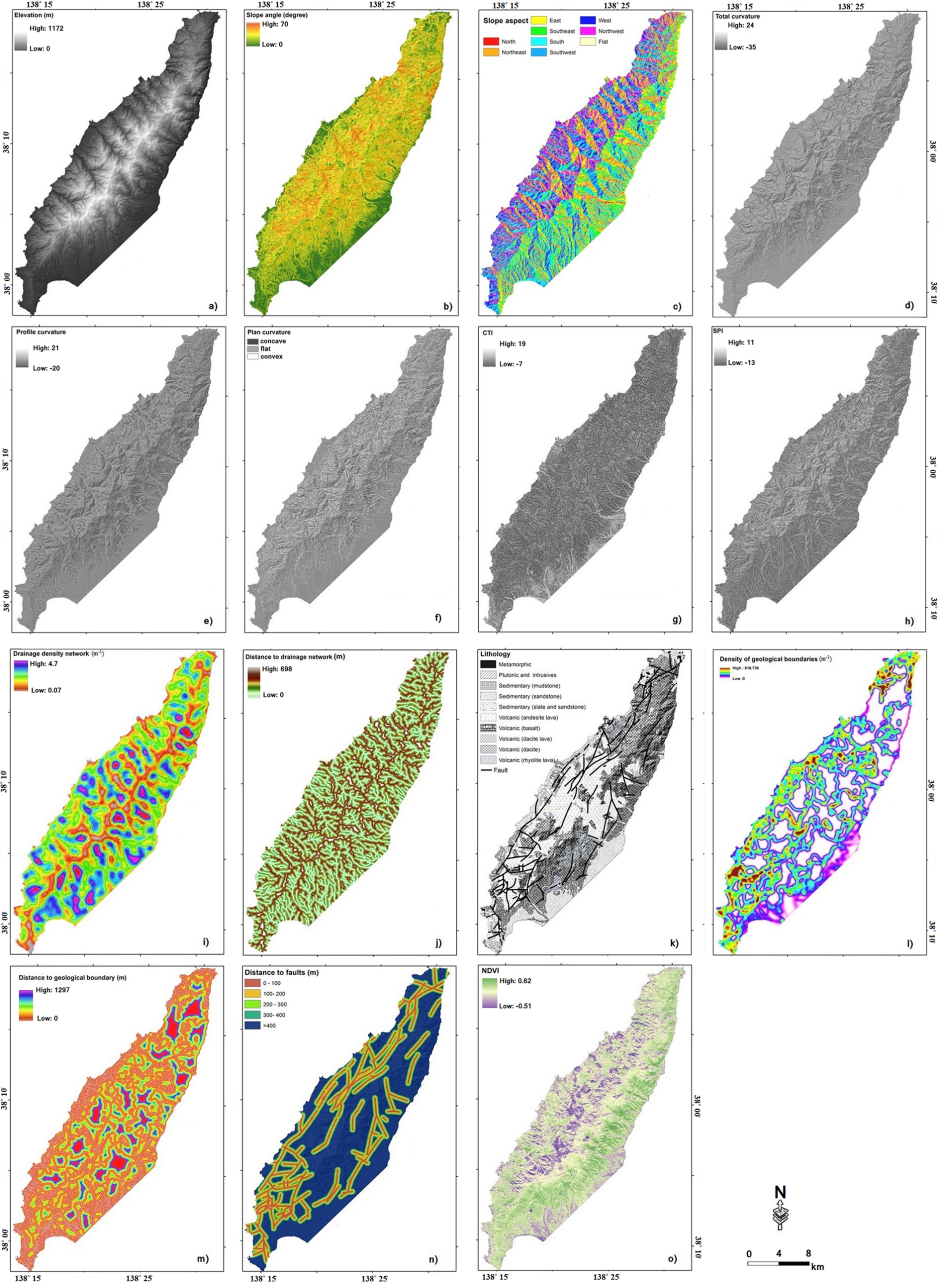
Landslide causative factors: a) elevation, b) slope angle, c) slope aspect, d) total curvature, e) profile curvature, f) plan curvature, g) CTI, h) SPI, i) drainage density (m^-1^), (j) distance from drainage networks, k) lithology, l) density of geological boundaries, m) distance to geological boundaries, n) distance to faults, and o) NDVI.

Landslides occurrence are influenced by the interaction of topographic, hydrological and geological factors [16,30], therefore, the selection of the causative factors is considered to be a fundamental step in the susceptibility modeling. In this study, based on analysis of the landslide inventory map and the underlying geo-morphometric conditions [10,44], a total of fifteen landslide-causative factors (Fig 5) commonly found in literature were firstly derived. These fifteen factors were extracted from their respective spatial database (Table 1). The source data for the landslide causative factors may vary in their scale and affect the accuracy of landslide susceptibility models [45]. To be commensurate with the diversity of the data source and difference in the scales, we converted all the factors to a raster format with a resolution of 10 m that corresponds the DEM resolution.

#### 2.3.1 Morphometric factors

The 10 m digital elevation model (DEM) obtained from the Geospatial Information Authority of Japan (GSI) were used to derive elevation, slope angle, slope aspect, total curvature, profile curvature, plan curvature, compound topographic index (*CTI*) and stream power index (*SPI*) using ArcGIS 10.2 software. The detailed classes and maps of these factors are shown in Fig 5.

Elevation is widely used for the assessment of landslide susceptibility. The variation in elevation may be related to different environmental settings such as vegetation types and rainfall [46]. Slope angle is typically considered to be one of the influential factor for landslide modeling because it controls the shear forces acting on hill slopes [29,47,48]. Slope aspect, that relates to sunlight exposure and drying winds control the soil moisture were also considered an important factor in landslide studies [17]. Total curvature is defined as the change in slope along a small arc of the curve. The profile curvature is the curvature in the down slope direction, while the plan curvature is the curvature of the topographic contours. All of them were found to influence the triggering of landslides [15]. Profile curvature influences the driving and resisting stresses within a landslide in the direction of motion and controls the change of velocity of mass movement flowing down the slope, whereas the plan curvature controls the convergence or divergence of landslide material and water in the direction of the landslide motion [49]. CTI and SPI are hydrological factors that are frequently used for the assessment of landslides [33]. According to Beven and Kirkby (1979) [50] and Gessler et al. (1995) [51], CTI and SPI could be calculated as follows:

CTI=ln(As/tanβ) (1)

SPI=As×tanβ (2)

where *As* is the specific catchment area per unit channel width orthogonal to the flow direction (m^2^/m) and *β* is the slope angle (degree).

#### 2.3.2 Geology-related factors

Lithology is considered one of the most influential factors in landslide susceptibility mapping because of its influence on the geo-mechanical characteristics of a terrain [30]. In this study, the lithology and faults were derived from the geology map at 1:200,000 scale obtained from the Geological Survey of Japan (GSJ). A total of ten lithological units were constructed: metamorphic, plutonic and intrusives, sedimentary (mudstone), sedimentary (sandstone), sedimentary (slate and sandstone), volcanic (andesite lava), volcanic (basalt), volcanic (dacite lava), volcanic (dacite), and volcanic (rhyolite lava).

It is found that geologic boundaries often relates to the rock strength. A high density of geologic boundary means lower stability and may lead to increase in landslide occurrences. Therefore the distance to geological boundaries also considered as a factor in this study. The closer the geological boundary, higher the probability for landslide occurrence [16]. All faults have been regarded as a critical factor in triggering landslide in tectonically active areas [29]. Additionally, the strength of fracturing and shearing stresses crucially influence the slope instability. Hence, distance to faults was also considered in this study to investigate the relationship between lineaments and landslide occurrence.

#### 2.3.3 Normalized difference vegetation index (NDVI)

The vegetation cover and the land use patterns often found to be of great influence in the landslide occurrences, because they relate to the anthropogenic interference on hill slopes [28,52]. The Normalized difference vegetation index (NDVI), an index of vegetation fraction was generated from the available cloud free ALOS (10 m resolution) satellite images which acquired on November 5th, 2006. NDVI is an indicator that reflects the amount of green vegetation [53] and can be computed using the following equation:

NDVI=(NIR−RED)/(NIR+RED) (3)

where, NIR and RED represents the spectral reflectance of near infrared and red bands of the electromagnetic spectrum, respectively. The values of NDVI vary from -1 to 1 and a higher value implies a denser green vegetation whereas lower values indicate sparse vegetation. High NDVI values are due to high concentration of chlorophyll that cause a relatively lower reflectance in the red band implying high stacking of leaves. Conversely, low NDVI values indicates less chlorophyll and less leaves [28].

## Methodology

### 3.1. Feature selection using Certainty Factor

The certainty factor (CF) is a rule based expert system method developed by Shortliffe and Buchanan (1975) [54] for the management of uncertainty in computational studies. CF provides probable favorability functions (FF) for integrating heterogeneous data [55] and can be calculated using the following functions:

$$CF=\{\begin{array}{c} \frac{PP_{\text{a}}-PP_{\text{s}}}{PP_{\text{a}}(1-PP_{\text{s}})}\;\text{if}\;PP_{\text{a}}\geq PP_{\text{s}}\\ \frac{PP_{\text{a}}-PP_{\text{s}}}{PP_{\text{s}}(1-PP_{\text{a}})}\;\text{if}\;PP_{\text{a}}<PP_{\text{s}} \end{array}$$ (4)

where PP_a_ is the conditional probability of landslides in class a and PP_s_ is the prior probability of total number of landslides in the study area.

The CF values range between -1 and 1, and it indicates a measure of belief and disbelief [37]. A positive value measures decreasing uncertainty whereas negative values imply an increasing uncertainty of landslide occurrence. If CF value equals 0, no information on the certainty is indicated. Once the CF values for classes of the causative factors are obtained, these factors are then incorporated pairwise using the combination rule [36] as follows:

$$Z=\{\begin{array}{l} \;CF1+CF2-CF1CF2\;CF1,\;CF2\geq 0\\ \;CF1+CF2+CF1CF2\;CF1,\;CF2<0\\ \frac{CF1+CF2}{1-\text{min}\left(\left|CF1\right|,\;\left|CF2\right|\right)}\;CF1,\;CF2,\;\text{opposite signs} \end{array}$$ (5)

The pairwise combination is carried out until all the CF layers are brought together. The causative factors are optimized by computing the Z values. If the Z values are positive, we regard those factors have high relationship with landslide occurrence.

Based on the range of CF values, feature weights were obtained. The weights are estimated as the sum of the ratio computed relative causative factors that provides a measurement of certainty in forecasting the landslides [36]. Based on the results, CF weights were then categorized into six classes. The description of the six classes is shown in Table 2.

**Table 2:** CF weights classification according to the range of CF values.

Code	Range	Description
1	−1.0–−0.09	Extremely low certainty
2	−0.09–0.09	Uncertainty
3	0.09–0.2	Low certainty
4	0.2–0.5	Medium certainty
5	0.5–0.8	High certainty
6	0.8–1.0	Extremely high certainty

### 3.2. Statistical index

The statistical index method (SI) proposed by Van Westen et al. (1997) is based on the assessment of correlation of the landslide inventory map and causative factors. In SI models, the weight for each class of the landslide causative factors was firstly determined. Landslide susceptibility indexes were then obtained by summing up the weights.

The weight (*W*_*i*_) of each class *i* is defined as the natural logarithm of the landslide density in the class over the landslide density in the factor map as follows[56]:

Wi=ln(DensClassDensMap)=ln(Npix(Si)Npix(Ni)∑Npix(Si)∑Npix(Ni)) (6)

where *W*_*i*_ is the weight given to a certain parameter class (e.g., lithology and slope aspect); D*ensClass* is the landslide density within the parameter class; *DensMap* is the landslide density of the entire factor map for all classes; *Npix(Si)* is the number of landslide pixels in a certain class; *Npix(Ni)* is the total number of pixels in all classes.

### 3.3. Binary Logistic regression

Binary logistic regression is one of the most frequently used multivariate analysis methods for creating landslide susceptibility maps. The LR approach is useful for situations in which one want to be able to predict the presence or absence of a characteristic outcome from a set of predictor variables [10,38]. The purpose of LR is thus to simulate the relationships between a dependent variable and multiple independent parameters [29]. The merit of LR is that it does not compulsorily require a normal distribution data. Additionally, both continuous and discrete data types can be used as an input for the LR model.

The dependent variable (Y) in the LR method is a function of the probability and can be computed as follows [57]:

$$P(\text{Y}=1|x)=\frac{\text{exp}(\sum bx)}{1+\text{exp}(\sum bx)}x$$ (7)

where *P* is the estimated probability of landslide occurrence and ranges from 0 to 1; Y is an indicator variable, *X* is the independent variables (landslide causative factors), X = (x_0_, x_1_, x_2_, … x_*n*_), x_0_ = 1; *b* is regression coefficient.

To linearize the mentioned method as well as remove the 0/1 boundaries for the original dependent variable, the estimated *P* probability is transformed by the following formula:

$$P^{'}=\text{ln}(\frac{P}{1-P})$$ (8)

The alteration is referred to as the logit transformation. Theoretically, the logit transformation of binary data can ensure that the dependent variable is continuous and the logit transformation is boundless. Moreover, it can ensure that the probability surface will be continuous within the range [0, 1]. Using the logit transformations, the standard linear regression models can be obtained as follows:

$$P^{'}=\text{ln}(\frac{P}{1-P})={\text{b}}_{0}+{\text{b}}_{1}\;x_{1}+{\text{b}}_{2}\;x_{2}+\ldots +{\text{b}}_{n}\;x_{n}+\varepsilon$$ (9)

where, b_0_ is the constant or intercept of the formula, *b*_1_, *b*_2_, … *b*_*n*_ represents the slope coefficients of the independent parameters, x_1_, x_2_, … x_*n*_ in the logistic regression and *ε* is standard error.

The LR model mainly involves five steps in generating the LSM models: 1) pre-selection of parameters based on the analysis of the spatial distribution; 2) selection of statistically significant parameters via a p-value significance test; 3) The LR model with these parameters has to pass the significance test (via the goodness of fit by inputting a parameter or eliminating a parameter); 4) evaluation of the multicollinearity among the parameters (diagnosis via two indicators, namely, tolerance <0.1 and variance inflation factor >5); 5) assessment of the accuracy in the model.

## Results

### 4.1. The relationship between landslide occurrence and causative factors

**Fig 6:**
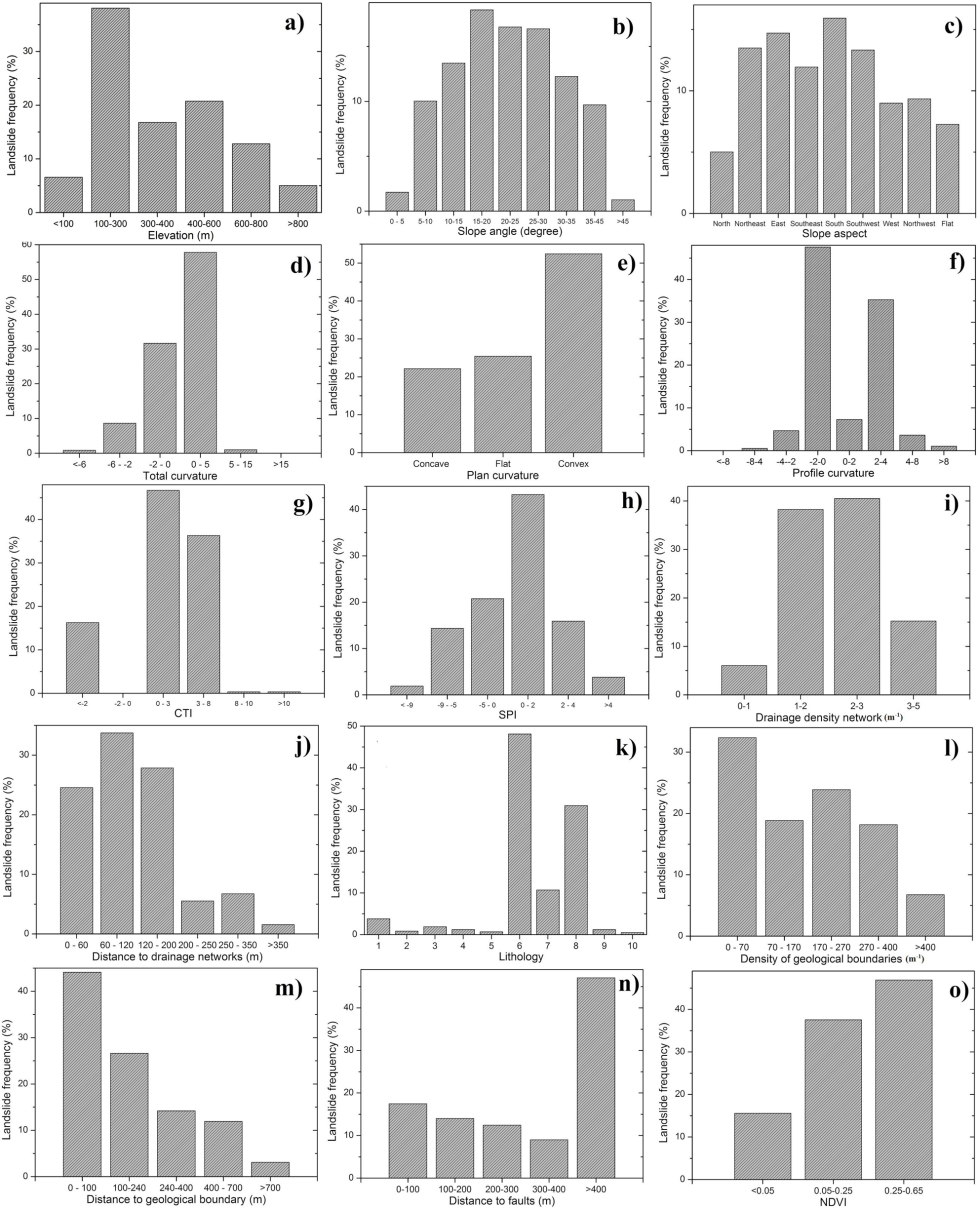
Correlations between landslide frequency and the causative factors.

Fig 6 shows the results of frequency analysis that explore the relationship between the landslide causative factors and landslide occurrence. It could be seen that the frequency of landslides is less than 10 percent at the elevation less than 100 m due to the gentle terrain characteristics (Fig 6A). At the intermediate elevation (100–300 m), the frequency of landslide occurrences is tending to increase, as slopes may be prone to slide due to the cover by the thin colluvium deposit. As expected, in the high elevation, the frequency increases. It is worth to point out that for elevation greater than 600 m, the areal extent of land is low and therefore the frequency of landslide occurrences is also lower. The correlation analysis between landslide occurrence and slope angle is shown in Fig 6B. It could be observed that gentle slopes have a low landslide frequency because of the lower shear stress at the slope angles 0–10° (Fig 6B). It is obvious that the landslide frequency increases for slope angles 15–35°. Followed by this a decrease of landslide occurrences at > 45° slope category was observed.

It is believed that slope angle and aspect may affects the vegetation patterns in the region. It may influence the soil strength and in some cases makes it susceptible to landslides. It is observed that the landslide frequency in a north-direction slope is relatively low, and it increases with the orientation angle, reaching the maximum on the south-direction slope, and then decreases (Fig 6C). Fig 6D shows that landslides mostly occurred at 0–5 category for the total curvature, while for the profile curvature landslides frequently occurred at the -2–0 category followed by the 2–4 group (Fig 6F). For the plan curvature (Fig 6E), the landslides usually occurred in the concave space because it increases the moisture content of the soil and leads to slope failure. But in this study, most landslides occurred in the convex space. This is possibly because the mountain ridges in Osado tend to collapse due to the local tectonics that causes higher ground acceleration.

For the hydrological factors CTI and SPI, landslides mostly occurred at 0–3 and at 0–2 respectively (Fig 6G–6H). It is noted that increasing density in the drainage network causes increasing occurrences of landslide frequencies. However, in this study landslides frequently occurred at 1–3 m^-1^and then decreased (Fig 6I). For the distance to drainage networks, landslide frequency reached the maximum at 60–120 m followed by 120–200 m (Fig 6J). It is attributed to the fact that topography change caused by gully erosion might affect the initiation of landslides.

As far as geological factors such as lithology, density of geological boundaries, distance to geological boundary, and distance to faults (Fig 6K–6N) are considered, they affect the strength and permeability that are associated with the slope failure. The results show that landslides mostly occurred in the volcanic (dacite) and volcanic (andesite lava) lithology. With respect to the density of geological boundaries, the landslide frequency mostly occurred at the 0–70 m^-1^, followed by 170–270 m^-1^ because higher geo-tectonic activity causes instability (Fig 6L). For the distance to a geological boundary, it is found that the weaker boundaries lead to instability. The landslide frequency decreases with increasing distance and has maximum at the <100 m (Fig 6M). Regarding the distance to faults, the results show that the majority of landslides falls into the category of the biggest distance to faults (>400 m) in the Fig 6N. The reason may relate to the parent material of the soil content and this class accounts for largest proportion of the area.

For the vegetation factor, landslide frequency normally occurred in the lower NDVI value (<0.05 and below) [58] because the roots of vegetation can retain the slope surface, especially for the shallow landslides. Nevertheless, in the case of relationship between landslide frequency and NDVI, the landslide mostly occurred in the high vegetation cover (NDVI > 0.25) because the shallow roots of vegetation seldom influence large landslide occurrence.

### 4.2. Feature selection using CF

The results of the correlation analysis between the landslide occurrence and causative factors are shown in Table 3. The result of CF analysis shows that the *Z* value is positive for slope angle (0.05), slope aspect (0.03), drainage density (0.34), lithology (0.3), distance to geological boundary (0.4) and distance to faults (0.35). It reveals that these six factors have positive relationships with the landslide occurrence. The *Z* value is negative for the other factors. Therefore, these six factors are selected to generate the landslide susceptibility mapping.

**Table 3:** Spatial relationship between the causative factors and landslide occurrence by the CF method and SI method.

Causative factors	Class	Percentage of domain (%)	No. of landslides	No. of landslide pixel	CF	Z	SI
Elevation (m)	<100	21.3266	38	8103	-0.6949	-0.4172	-1.5948
	100–300	30.6789	220	62288	0.1969		0.0811
	300–400	13.0918	97	31464	0.2232		0.2498
	400–600	17.7436	120	50504	0.1475		0.4189
	600–800	11.7973	74	29400	0.0797		0.286
	>800	5.3619	29	5460	-0.0652		-0.609
Slope angle (°)	0–5	11.7719	18	6317	-0.683	0.0514	-1.247
	5–10	9.0556	58	26860	0.2563		0.4627
	10–15	8.9907	103	27721	0.5897		0.3197
	15–20	10.3467	126	31045	0.6147		0.4742
	20–25	11.9734	86	23115	0.3382		0.0332
	25–30	13.3891	89	28873	0.2839		0.1439
	30–35	13.9796	47	21260	-0.2997		-0.2053
	35–45	18.0504	45	18667	-0.4818		-0.591
	>45	2.4425	6	3361	-0.4895		-0.3054
Slope aspect	North	5.7447	32	10672	-0.0368	0.0295	-0.0078
	Northeast	12.6420	65	25359	-0.1119		0.069
	East	12.8910	82	25931	0.0927		0.0718
	Southeast	12.5850	80	24548	0.0921		0.044
	South	13.4147	92	27897	0.1596		0.1051
	Southwest	14.6818	74	23033	-0.1296		-0.0167
	West	11.8353	59	20342	-0.1393		-0.0855
	Northwest	10.6724	54	19635	-0.1262		-0.0175
	Flat	5.5332	40	10802	0.2034		0.0419
Total curvature	<-6	21.3266	5	3321	-0.96	-1	-2.4868
	-6–-2	30.6789	50	23168	-0.721		-0.9079
	-2–0	13.0918	183	64542	0.5952		0.9682
	0–5	17.7436	334	93753	0.7033		1.0375
	5–15	11.7973	6	2433	-0.9132		-2.2059
	>15	5.3619	0	2	-1		-8.521
Profile curvature	<-8	0.0254	0	7	-1	-1	-1.91678
	-8–4	0.8660	3	1189	-0.4042		-0.3101
	-4–2	5.8053	27	9310	-0.1976		-0.1547
	-2–0	40.0014	275	76347	0.1616		0.0192
	0–2	9.0359	42	11779	-0.1981		-0.362
	2–4	36.7382	204	75185	-0.0398		0.089
	4–8	5.8333	21	10719	-0.3806		-0.0186
	>8	1.6943	6	2690	-0.3908		-0.1648
Plan curvature	Concave	8.0198	34	15730	-0.2694	-0.2237	0.0465
	Flat	36.2449	212	69796	0.012		0.0282
	Convex	55.7353	332	101693	0.0301		-0.0258
CTI	<-2	14.4751	94	23738	0.1108	-1	-0.1338
	-2–0	0.3435	0	480	-1		-0.2926
	0–3	52.9836	270	94694	-0.1199		-0.0464
	3–8	29.6776	210	62790	0.1859		0.1223
	8–10	1.4678	2	3866	-0.7669		0.3414
	>10	1.0525	2	1651	-0.6745		-0.1769
SPI	<-9	3.4962	11	3023	-0.4593	-0.6077	-0.7725
	-9–-5	11.0713	83	20789	0.2324		0.003
	-5–0	26.5513	120	39004	-0.2206		-0.2425
	0–2	42.3751	250	84561	0.0206		0.0638
	2–4	12.5642	92	28670	0.2138		0.1979
	4–12	3.9418	22	11172	-0.0349		0.4147
Drainage density (m^-1^)	0–1	10.3116	35	10261	-0.4164	0.3444	-0.6311
	1–2	40.9824	221	70163	-0.068		-0.0885
	2–3	37.9238	234	78979	0.0642		0.1074
	3–5	10.7822	88	27907	0.2962		0.3248
Distance to drainage networks (m)	0–60	25.4378	142	64448	-0.0347	-0.818	0.302
	60–120	22.9935	208	49926	0.3664		0.1477
	120–200	23.6065	148	39592	0.0792		-0.1105
	200–250	11.1821	32	15262	-0.5086		-0.3165
	250–350	12.8205	39	15250	-0.4774		-0.4541
	>350	3.9595	9	2832	-0.6103		-0.9627
Lithology	1. Sedimentary (sandstone)	15.4136	27	8082	-0.7	0.2977	-1.273
	2. Sedimentary (mudstone)	3.9514	11	1500	-0.522		-1.596
	3. Plutonic and intrusives	2.2348	15	5005	0.1411		0.1789
	4.Volcanic (basalt)	0.6317	6	1207	0.3974		0.02
	5. Volcanic (rhyolite lava)	0.6294	3	281	-0.1773		-1.4338
	6. Volcanic (dacite)	38.8175	285	95356	0.2161		0.2713
	7.Volcanic (dacite lava)	5.7499	52	12766	0.3664		0.1702
	8. Volcanic (andesite lava)	30.9204	162	60295	-0.0946		0.0407
	9.Sedimentary (slate and sandstone)	1.0510	7	1308	0.1343		-0.4084
	10.Metamorphic	0.6002	10	1430	0.6629		0.241
Density of geological boundaries (m^-1^)	0–70	30.2616	184	65069	0.0501	-0.0279	0.1385
	70–170	21.9598	112	41448	-0.1192		0.0081
	170–270	23.5557	138	48197	0.0136		0.0888
	270–400	17.4113	105	23067	0.0422		-0.3458
	>400	6.8116	39	9498	-0.0096		-0.2947
Distance to geological boundary (m)	0–100	48.5691	255	74525	-0.0929	0.3989	-0.199
	100–240	25.6755	154	54091	0.0369		0.118
	240–400	13.7894	82	30935	0.0284		0.1809
	400–700	9.7160	69	21069	0.1889		0.1469
	>700	2.2500	18	6659	0.2816		0.0009
Distance to faults (m)	0–100	13.1941	101	23269	0.2485	0.3481	-0.0601
	100–200	11.8204	81	20520	0.1588		-0.0759
	200–300	10.6687	72	16936	0.1456		-0.1653
	300–400	9.2542	52	14905	-0.0283		-0.1508
	>400	55.0625	272	111638	-0.1473		0.0793
NDVI	<0.05	24.4313	89	24965	-0.3749	-0.2216	-0.2082
	0.05–0.25	37.6047	218	49489	0.003		0.0448
	0.25–0.65	37.9640	271	51402	0.1945		0.0733

A detailed analysis shows that slope angle has the highest influence on the slope stability. CF values are positive at slopes from 5° -30° (Table 3). The percentage of landslide occurrence at the slope class 10° -15°, 15° -20°, and 25° -30° are 17.82%, 21.79%, and 15.4%, respectively. The results indicate that the landslide occurrence increases with an increasing slope angle up to 20°, and then it decreases. The conclusion is in agreement with the landslide frequency in Fig 6C. In the case of slope aspect, landslides mostly occurred along east, southeast, and south facing slopes with positive CF values from 0.09 to 0.15. The highest percentage of landslides with the maximum CF value (0.15), 15.9% occurred along the southern slopes, followed by the east slopes (14.19%). The snow in the study area is normally blown out by the wind from the northwest; therefore, southeast slopes accumulate snow that during the snow melting is causing slides to occur. With respect to the drainage density, it shows positive values for the classes 2–3 and 3–5. The maximum positive CF value of 0.3 is with the 3–5 class. The highest percentage of landslide occurrence is 40.48% at 2–3 class. In the case of lithology, the results show that six lithology classes have positive CF values. The highest percentage of landslides in the lithology class (volcanic-dacite) is 49.31% with a CF value of 0.22. It could be observed that > 50% of the landslides occurred along the margins of dacite and dacite lava. These lavas once covered by ocean may transform into pelitic rocks that further may change to materials rich in smectite clay and become subject to sliding. The class “Distance to geological boundary” shows positive values for classes >100 m in Table 3, however, the highest percentage of landslides occurred for less than 100m. It indicates that the closer to geological boundary, the more occurrences of landslides. The distance to faults shows a positive value for the classes, 0–100 m, 100–200 m, and 200–300 m and then the CF values become negative over 300 m. The maximum CF value is 0.25 at 0–100 m.

### 4.3. Landslide susceptibility mapping using SI method

**Fig 7:**
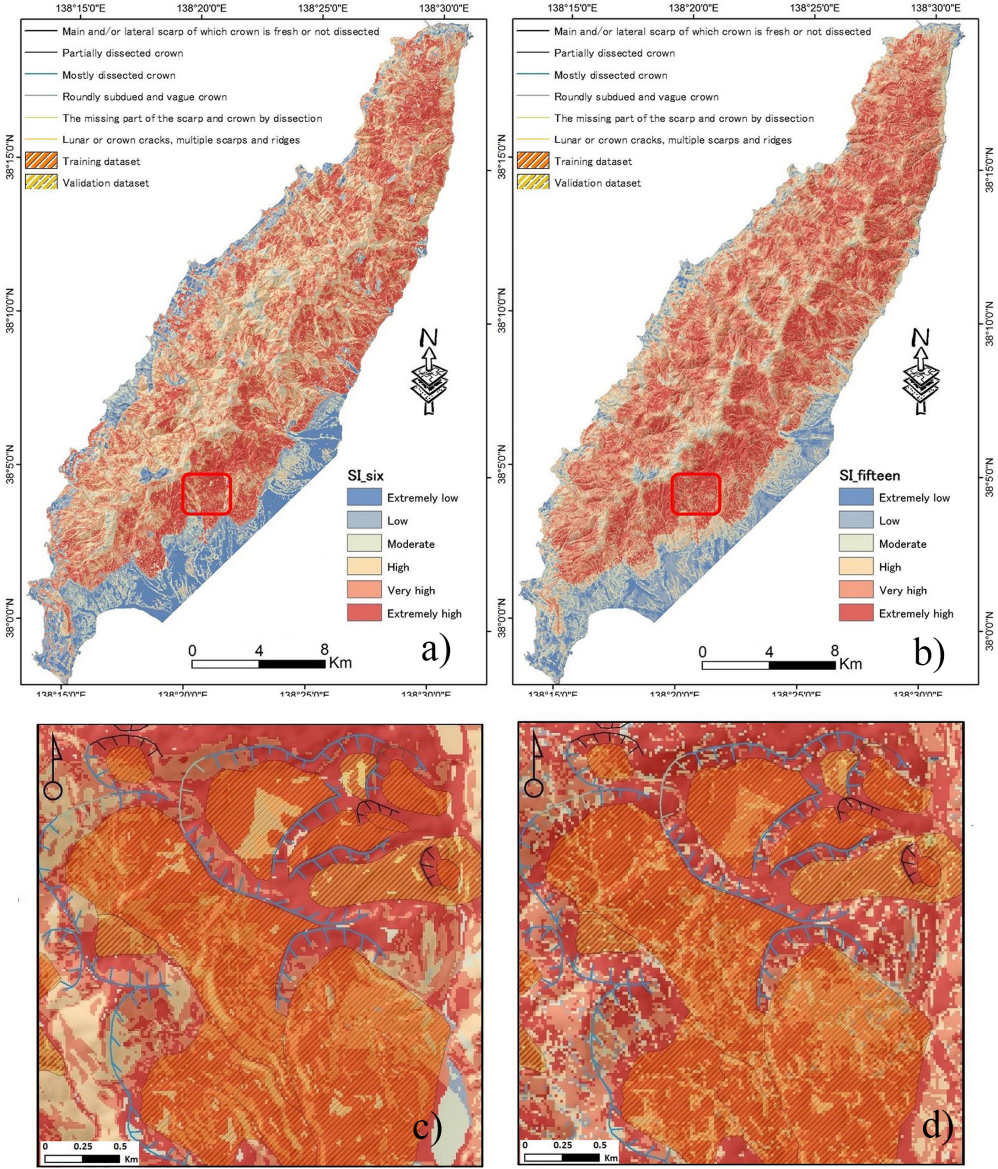
LSM maps generated by the SI method using: a) six factors, and b) fifteen factors. The maps (c) and (d) are enlarged views of the LSM maps (red color boundary shown in (a) and (b)).

**Fig 8:**
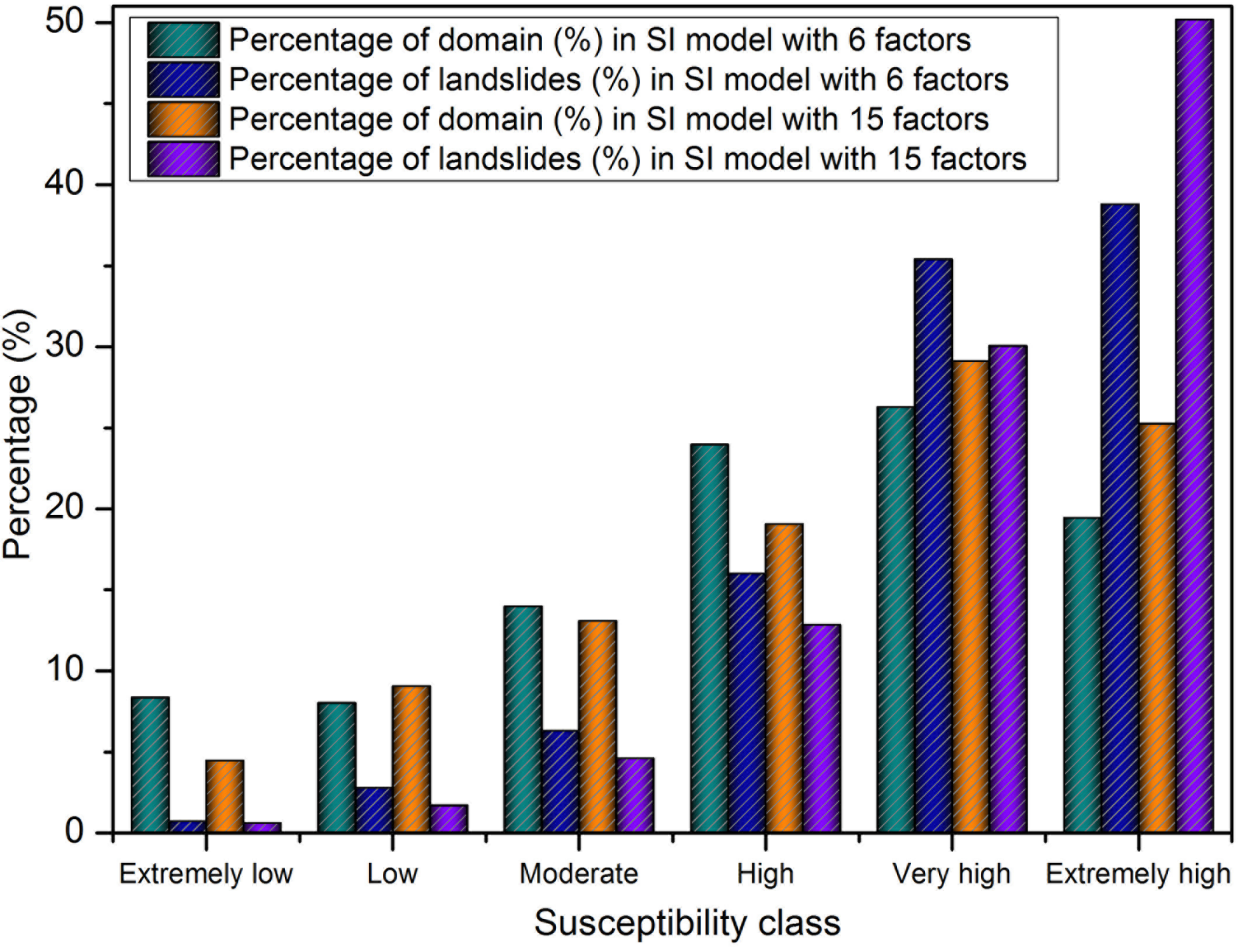
Comparison of landslide susceptibility class obtained from the SI model.

The correlation between the landslide occurrence and causative factors using SI is represented in Table 3. Two landslide susceptibility maps were generated: (i) using the six selected factors (CF value > 0) and (ii) using originally selected fifteen factors. The results that indicate the spatial probability of landslide occurrence is shown in Fig 7. Based on the natural breaks inherent in the data, the susceptible level is eventually divided into six classes; i.e., extremely low, low, moderate, high, very high and extremely high. Table 4 shows the boundary classes for two susceptibility maps. It can be noticed from the visual observation that there are much more red color areas in Fig 7B, whereas there are more dark blue areas in Fig 7A. In the figures, black lines denotes main scarp and the blue lines denotes dissected crown. Fig 8 shows that 90.18% of the total number of landslides occurred in the 69.66% of the area classified as having high, very high and extremely high susceptibility using the optimized six factors, while 73.41% of the total number of landslides occurred in the 93.1% of the area classified as having high, very high and extremely high susceptibility using the original fifteen factors.

**Table 4:** The classes used for susceptibility maps.

Susceptibility class	SI method		LR method	
	fifteen factors	six factors	fifteen factors	six factors
Extremely low	−12.31–-3.41	−3.83–−2.07	0.00–0.13	0.03–0.20
Low	−3.41–-2.17	−2.07–−1.20	0.13–0.32	0.20–0.38
Moderate	−2.17–-0.94	−1.20–−0.57	0.32–0.51	0.38–0.51
High	−0.94–0.23	−0.57–−0.04	0.51–0.67	0.51–0.64
Very high	0.23–1.33	−0.04–0.44	0.67–0.81	0.64–0.75
Extremly high	1.33–4.26	0.44–1.85	0.81–0.98	0.75–0.91

According to Table 3, the slope angle class (15° -20°) with the highest SI value of 0.47 is most susceptible, having the highest percentage of landslide occurrence 16.58%. The results indicate that the landslide susceptibility gradually increases with increasing slope angle and then it drops after 35°. This result is similar to that of CF.

Landslide susceptibility map shows that the areas along northeast, east, southeast and south facing slopes are highly susceptible. The highest percentage of landslides with the maximum SI value (0.1) is 14.9% along the southern slopes, followed by the 13.85% for the east facing slopes. This also agree with the results obtained from CF.

With an increase in drainage density, the SI values are amplified suggesting that landslides are more prone to occur at the classes of SI value 2–3 m^-1^and 3–5 m^-1^. The highest percentage of landslide occurrence inside this class is 42.19%. This result is also in agreement with those obtained from the CF analysis.

With respect to lithology, the results also display that six lithology classes (similar with CF) have a high relationship with landslide occurrence. The highest percentage of landslides among the lithology class (volcanic-dacite) is 50.93% with a maximum SI value of 0.27. It is perceived that landslide occurrence along the margins of dacite and dacite lava are greater than 50%. The distance to geological boundary indicates that classes >100 m have a high probability of landslide occurrence (Table 3). The highest percentage of landslides for the class, occurred at less than 100 m and is 39.81%. The distance to faults exhibits negative SI values for the classes, 0–100 m, 100–200 m, and 200–300 m, and 300–400m and then the SI values become positive after 400 m.

### 4.4. Landslide susceptibility mapping using LR model

In this study, the forward stepwise logistic regression approach was used to incorporate predictor variables with a main contribution to the presence of landslides, using the SPSS 20. In the training dataset 578 landslides represented the presence of landslide points and were assigned the value 1. In agreement with the equal proportions of landslide and non-landslide, the same number of non-landslide points were randomly sampled from the landslide-free area and assigned the value 0.

The result is shown in Table 5. It shows that all the causative factors have a P-value less than 0.1, indicating a statistical correlation between factors and the susceptibility of landslides at the 90% confidence level [29]. The interpretation of the logistic regression coefficient for each causative factor shows that elevation, slope angle, slope aspect, total curvature, SPI, drainage density, lithology, distance to drainage network, distance of geological boundary, and NDVI have positive values (Table 5). Distance to drainage network has the highest value (1.7), followed by slope angle (1.2). On the other hand, the excluded factors have a negative effect on landslide occurrence.

**Table 5:** Coefficients, statistics of the factors (S.E.-standard error, VIF- variance inflation factor) and the multi-collinearity diagnosis indexes for variables used in the logistic regression equation.

Causative factors	Coefficient (B)	S.E.	P-value	Exp (B)	Collinearity Statistics	
					Tolerance	VIF
Elevation	0.956	0.133	0	2.601	1	1
Slope angle	1.209	0.193	0	3.350	0.977	1.023
Slope aspect	0.283	1.574	0.085	1.327	1	1
Total curvature	0.308	0.121	0.011	1.361	1	1
Profile curvature	-0.756	1.695	0.065	0.470	0.994	1.006
Plan curvature	-1.186	3.666	0.074	0.305	0.997	1.003
CTI	-0.23	0.985	0.081	0.795	0.987	1.013
SPI	0.699	0.491	0.015	2.012	0.995	1.005
Drainage density	0.123	0.429	0.077	1.131	0.999	1.001
Distance to drainage networks	1.706	0.329	0	5.507	0.997	1.003
Lithology	0.879	1.564	0.023	2.408	0.994	1.006
Density of geological boundaries	-0.045	0.538	0.093	0.956	0.999	1.001
Distance of geological boundaries	0.853	0.571	0.013	2.347	0.973	1.028
Distance to faults	-0.441	0.888	0.061	0.643	0.988	1.012
NDVI	0.283	0.839	0.073	1.327	0.992	1.008
Constant	0.791	0.121	0.000	2.206		

Additionally, it is necessary to examine the effect of correlation because logistic regression is sensitive to collinearity among the independent variables. The variance inflation factor (VIF) and tolerance (TOL) are widely used indexes of the degree of multi-collinearity. A VIF value greater than or equal to 5 and a TOL value less than 0.2 indicates a serious multi-collinearity problem [59]. In this study, both of these indexes were calculated (Table 5), the maximum VIF and minimum TOL were 1.028 and 0.973, respectively. Therefore, there is no multi-collinearity between these variables in the study.

**Fig 9:**
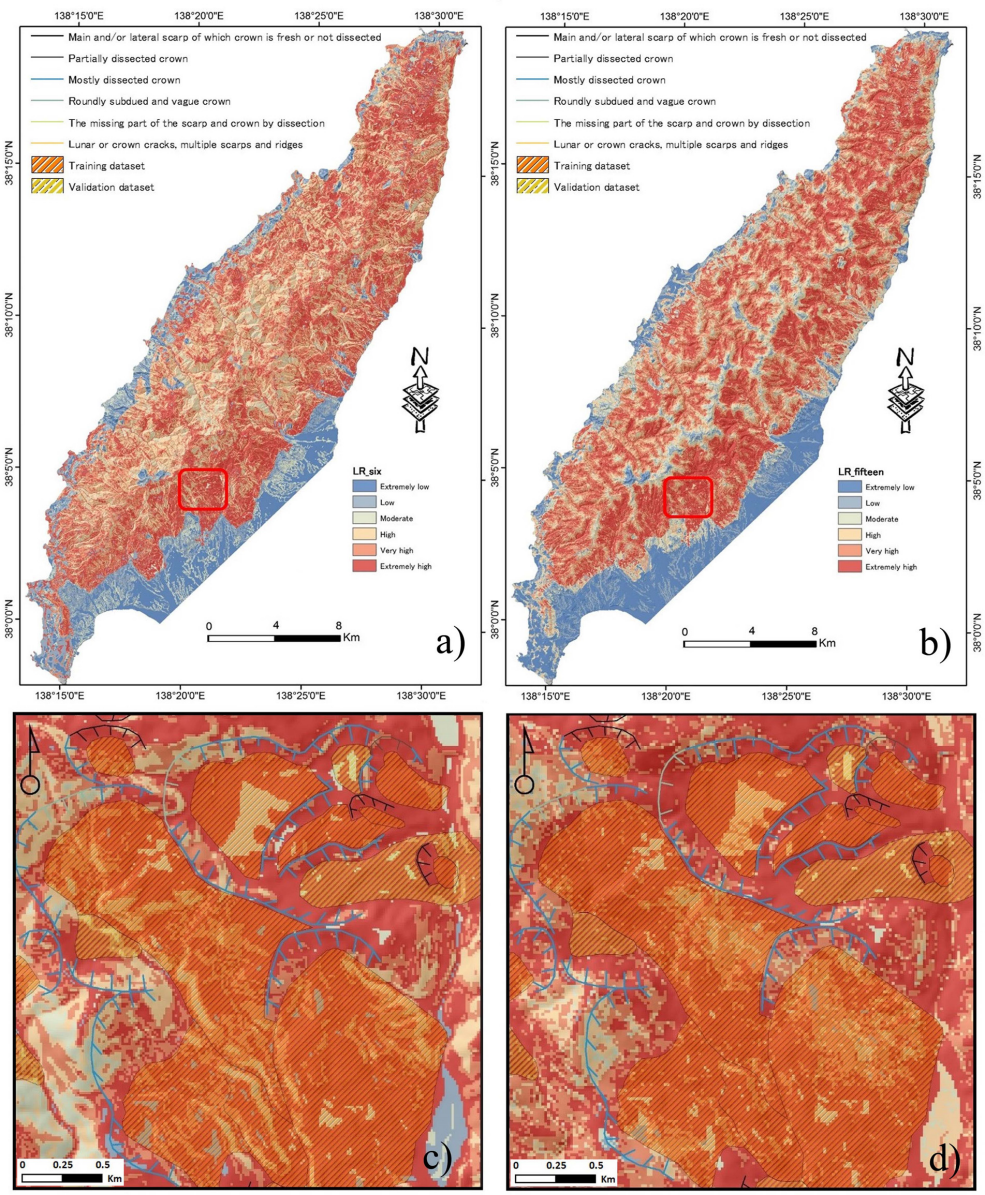
LSM maps generated by the LR model using: a) six factors, and b) fifteen factors. The maps (c) and (d) are enlarged views of the LSM maps (red color boundary shown in (a) and (b)).

**Fig 10:**
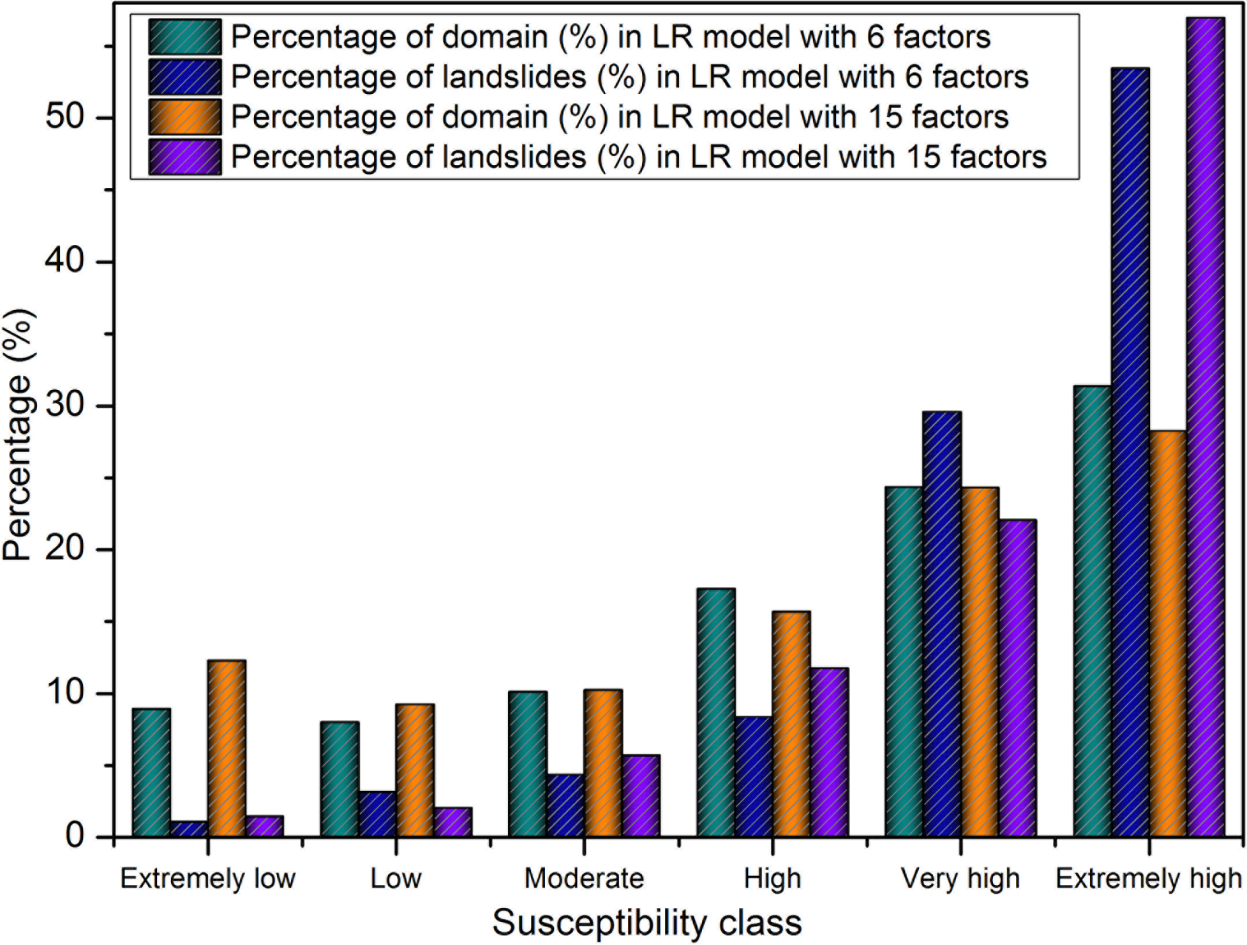
Comparison of landslide susceptibility class obtained from the LR model.

Lastly, the regression coefficients of the predictors were imported to generate the landslide susceptibility map (Fig 9) in GIS by using the Eqs (7) and (9). The two maps of classes are also both applied the natural break classification to divide the boundaries of each class (Table 4). Fig 10 shows that 91.39% of the total landslides took place in the 72.96% of the area classified as high, very high and extremely high using the optimized six factors, while 68.23% of the total landslides occurred in the 90.79% of the high, very high and extremely high area using the original fifteen factors.

### 4.5. Accuracy assessment of susceptibility maps

LSM results can be validated using the known landslide locations. Accuracy assessment was performed by comparing the existing landslide spatial distribution data, that was not included in the data used to create the LSM maps. The area under curve (AUC) is a useful indicator to validate the prediction performance of the model. An area of 1 in the AUC represents a perfect test; an area of 0.5 represents a worthless test. In this study, both the training (70% of 825 landslide polygons) and validation (the rest 30% of 825 landslide polygons) datasets were selected to assess the models. The training data was used for the LSM success rate and the validation data for the prediction rate. To obtain both values, the landslide susceptibility index (LSI) values of all cells were sorted in descending order. Then the ordered grid values of the LSI were categorized into 100 classes with 1% cumulative intervals, for which the cumulative percentage of landslide occurrence in the classes was calculated to get the AUC.

**Fig 11:**
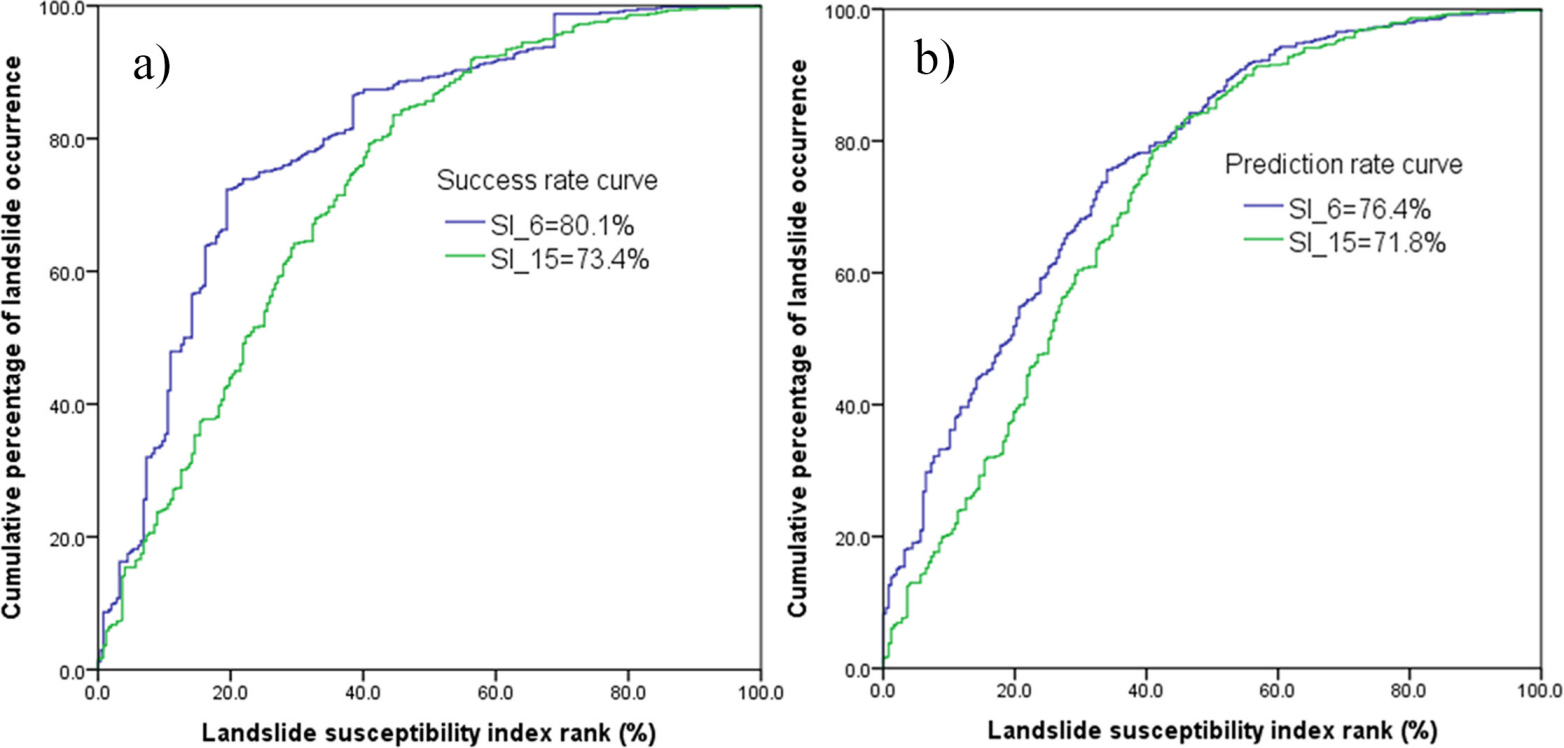
Area under curve (AUC) represents: a) success rate, and b) prediction rate using SI method.

**Fig 12:**
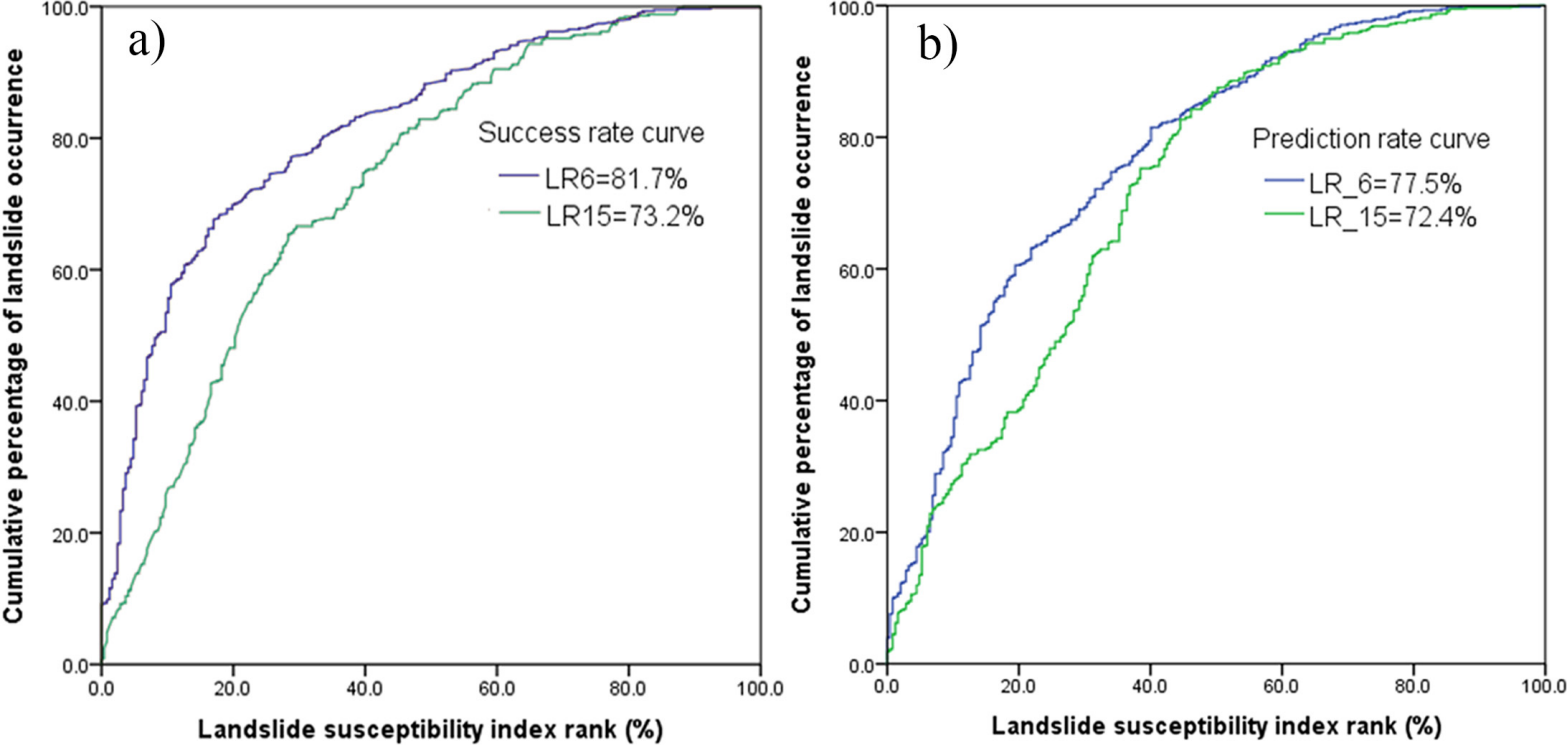
Area under curve (AUC) represents: a) success rate, and b) prediction rate using LR model.

From Fig 11, it can be seen that for the SI method the AUC value of the success rate curve (80.1%) using six factors is higher than for the model using all fifteen factors (73.4%). For the prediction rate curve, the results have a similar trend as the success rate curve. In the LR model, the AUC value of the success rate curve (81.7%) using six factors is higher than that of (73.2%) using all fifteen factors as shown in Fig 12. At the same time, the prediction rate has similar results as the success rate. Hence, it is observed that using the six factors give higher accuracy than that of using all the fifteen factors. Additionally, compared with the SI method, LR has a slightly higher accuracy in both success rate and predication rate.

## Discussion

The results presented here deals with two main topics: (i) a procedure to select the best landslide causative factors, and (ii) mapping landslide susceptibility in the Osado region based on the selected causative factors using statistical index and logistic regression.

A prior knowledge of appropriate causative factors related to landslide events is required to map landslide susceptibility [39]. Several studies [30,60] in the past have shown that a manual selection of the causative factors by a subject specialist was considered the best approach, but it is rather subjective. Indeed, so far there is no general criteria or guidelines available on how to identify and select the number of landslide causative factors. Due to this fact, numerous scholars have used a varied number of different causative factors to produce the landslide hazard map. In literatures, it is found that sometimes 20–60 factors have been used for building discriminant susceptibility models [39]. Nevertheless, most frequently 10–15 factors were used based on availability and accessibility of information [60]. Hence, it is possible to narrow down the factors based on the knowledge of triggering mechanism involved. For instance, in earthquake-induced landslides, the triggering factors associated are no way related to precipitation and their varieties, but are linked to ground acceleration and intensity. In such case, it is a common understanding that one can easily omit those unnecessary factors in the analysis. However, when the triggering mechanism is unknown, where the landslide inventory database were created from multiple imageries in different period of time, the screening out process requires statistical or computational models. Lee et al (2008) [60], on computing the standardized difference of causative factors, screened six factors out of 14 for landslide susceptibility mapping in the parts of Taiwan. Although this method includes less computation, it requires to categorize the data into landslide and non-landslide groups which is rather tedious. Similar statistical equations based on correlation or association indexes limit the predictive performances on multivariate models. On the other hand, Costanzo et al. (2012) [30] identified the factors based on the ranks associated with the factor’s expected contribution to the predictive skill of a multivariable model. Approaches adopting discriminant analysis and logistic regression on the forward selection of variables, however fail when most of the variables are statistically significant. For the same reason, this study did not consider the stepwise LR model because we found most of the variables are significant in the statistical tests (P < 0.1, Table 5). As indicated in the results, none of the factors were screened with the stepwise LR model. Furthermore, stepwise LR model in landslide susceptibility assessment requires both landslide and non-landslide pixels in the calculation. The proposed model using CF eliminated these limitations because it used only landslide pixels in the computation, and hence is very fast. Prior definition of hazard classes is not required in CF approach and it also supplies advantage of rendering the definition of susceptible classes transparent. Moreover, the proposed model is a relatively straightforward method that allows the causative factors to be ranked according to their certainty values in the range between -1 to 1. It is assumed that positive CF values have a strong influence on the landslide occurrence, and vice versa. As shown in the result, and the criterion discussed in the section 3.1, six causative factors were finally identified and they were ranked from 1 to 6 based on their CF values; where 1 indicates “low certainty” and 6 indicates “high certainty”. We believe, CF based factor screening process for the identification of the most determinant factors is an important step in the landslide hazard mapping.

The identified six landslide causative factors (slope angle, aspect, drainage density, lithology, and distance to geological boundary and distance to faults), all have high correlation with landslide occurrence. Moreover, the results were also validated by the success rate and predication rate. It is found that the LSM produced from the six factors always have higher accuracy than that of the original fifteen factors in both the statistical index and logistic regression models. The results demonstrated that a larger number of causative factors does not necessarily obtain a better landslide predictability map. This is probably either because of the data redundancy or spatial self-correlation with the study area. For instance, one of the causative factors, NDVI has no significant effect on the landslide occurrence in this study, as most of the landslides were large. Relatively short roots of the vegetation cover do not considerably influence large landslides and should be obvious to our understandings. In addition, geology and faults may have a positive influence on triggering deep-seated landslides. As demonstrated in section 4.1, landslide activity is mostly concentrated in the lithologies dominated by volcanic dacites, and volcanic andesites, followed by volcanic dacite lava and sandstone. Volcanic dacite and andesite are characterized by a high silica and alumina content and low in potash, they generally have relatively low shear strength and are strongly fractured, resulting in most concentrated landsliding in these rocks. Furthermore, slopes consisting of these lithologies are relatively more steep and susceptible to failure (Fig 5K). Some authors [29,38] invoked faults as the triggering cause of many deep-seated landslides. Ayalew et al. (2005) reported presence of active faults in Sado Island that could potentially trigger landslides. This is in agreement with our results as confirmed from the CF analysis.

Although the method proposed in this study has not been tested at other sites, there are indications, which suggests its applicability to other landslide prone regions. Firstly, notwithstanding the fact that CF methods have seldom been used in identifying causative factors in landslide susceptibility mapping, they are used worldwide for managing uncertainty in rule-based systems. Because of their favorability functions to handle different data layers and the heterogeneity and uncertainty of the data, CF models are largely appreciated in slope stability studies [18,29,36,38]. Further, the causative factors used for successful preparation of landslide susceptibility mapping in Kosado region by Ayalew et al. (2005), is similar with the results obtained from this model. Although we selected fifteen factors initially, CF identified the major 6. In addition, the results obtained from both the training and testing data sets yield high accuracy.

## Conclusions

Landslide susceptibility mapping is essential to describe the propensity of a landslide in a susceptible area. This study demonstrates the usefulness of the certainty factor model in identifying the best fitted causative factors for landslide susceptibility mapping. Based on the CF model, six influencing factors with high correlation to landslide occurrence were selected from a set of fifteen factors. The LSM maps were then produced by applying both the SI and LR methods for the CF identified causative factors and the original set of factors. Both the success rate and prediction rate indicated for both the SI and LR methods that the six factors achieve better results than that of all fifteen factors. In addition, we noticed that the maps prepared from using six causative factors have much more homogeneous classes than the fifteen factors. Also, it is noted that the LR has slightly higher prediction performance (81.7%) than SI (80.1%). The proposed method provides a compact way to select the controlling factors of landslides in particular where data redundancy or scarcity is critical.

Finally, the results of such studies can provide helpful information for the disaster managers, for urban planners, and decision makers in the landslide-prone area. These maps can be helpful for them to select the suitable spatial locations to implement reconstruction strategies. They can use produced maps to avoid development in landslide threatened areas; the practice will represent the most efficient and economic way to decrease future damages and loss of lives in the local region.
